# Influence of Pb Bonding and Speciation on the Pyrolysis Products of Contaminated Biomass

**DOI:** 10.1021/acs.energyfuels.5c05968

**Published:** 2026-02-14

**Authors:** Davide Amato, Paola Giudicianni, Corinna Maria Grottola, Raffaele Ragucci

**Affiliations:** 518738 Istituto di Scienze e Tecnologie per l’Energia e la Mobilità Sostenibili (STEMS-CNR), Via Marconi 4, 80125 Naples, Italy

## Abstract

Phytoremediation is receiving increasing attention as an environmentally friendly remediation technique for contaminated soils, as it can target different contaminants, such as heavy metals. Phytoremediation processes produce large volumes of contaminated biomass that must be disposed of and possibly valorized. Among the possible treatments for heavy metal-contaminated biomass, a promising approach is to pyrolyze these biomasses. However, it must be considered that heavy metals can interact with the biomass pyrolysis decomposition pathways, resulting in variations in the yields and properties of pyrolysis products. In this work, the effects of lead (Pb) contamination on poplar biomass during slow pyrolysis were investigated. In particular, the focus of this paper is on the effect of the type of bonding of Pb with the biomass tissue, which is specific to the contamination type (authigenic or detrital), and on the effect of the chemical speciation of Pb. To study these aspects, poplar biomass was opportunely doped with lead acetate (Pb­(CH_3_COO)_2_) following different procedures aimed at simulating different bonds between Pb and biomass tissues. Moreover, to study the effect of Pb chemical speciation, poplar biomass was also doped with lead nitrate (Pb­(NO_3_)_2_). All the doped feedstocks, together with the parent biomass, were pyrolyzed under slow pyrolysis conditions at two pyrolysis temperatures (465 and 600 °C), and the obtained products, namely, biochar, bio-oil, and pyrolysis gases, were analyzed thoroughly. The obtained results show that the presence of Pb can indeed modify the pyrolysis pathways of lignocellulosic biomasses. The different bonding of Pb with biomass causes modifications in the yield of the liquid products. On the other hand, changing the Pb chemical speciation cause variations in the properties of all pyrolysis products. However, the extent of many Pb effects seems to be affected by the presence of inherent inorganics, such as alkali and alkali-earth metals (AAEMs).

## Graphical abstract

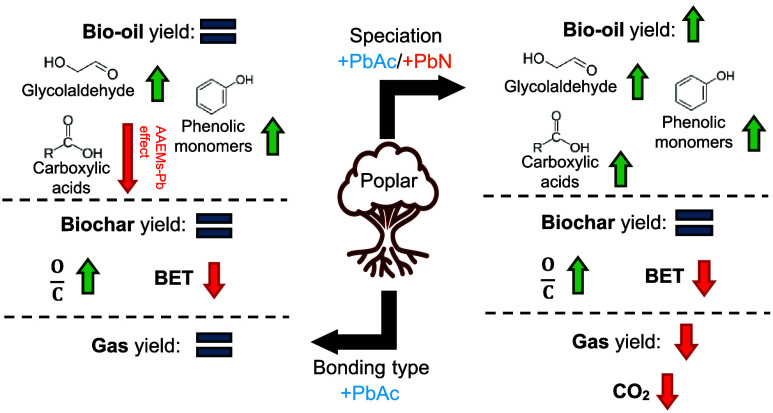

## 1 Introduction

Recently, phytoremediation diffusion has been increasingly used as a restoration technique for heavy metal-contaminated soils. Phytoremediation is based on the ability of some plants to take up heavy metals from the soil, confiscate them in their tissues (phytoextraction), and/or immobilize them in the soil itself (phytostabilization).^1^ If short rotation coppices (SRC) are used, this practice results in a large volume of contaminated biomass consisting of the aerial parts of the plants that could be annually harvested or pruned for further valorization as bioenergy. However, the safe valorization of contaminated biomasses and their economic relevance remain controversial due to the presence of potentially toxic elements (PTE), such as heavy metals. A promising treatment for heavy metal-contaminated biomass is pyrolysis,^2^ as by properly tuning the operational parameters of the process, it is possible to concentrate the contaminants in the solid residue (biochar),^3^ while also limiting their mobility.^4^ For this reason, most of the available literature studies focus on the final distribution of heavy metals in the pyrolysis products, which can be greatly influenced by both the adopted operational conditions^5^ and the pyrolysis plant configuration.^6^

However, the possible catalytic effect of heavy metals on biomass decomposition mechanisms has been underinvestigated, as well as their effect on the pyrolysis products′ yield and properties.^7^ Given the well-documented catalytic effect of lignocellulosic biomass inherent inorganics (e.g., alkali and earth alkali metals^8^), it is likely that heavy metals can exhibit catalytic effects as well, despite their low concentration. Indeed, the effect of heavy metals during pyrolysis has been explored for other kinds of feedstocks, such as animal residues or sludges.^9^ However, such results cannot be trivially transferred to lignocellulosic biomasses, given the different nature of the feedstock.

A review of the literature highlights several critical aspects.

- Unrealistic contamination levels: many studies consider doping concentrations far higher than those found in naturally contaminated biomasses,^10^ producing often contrasting results, possibly due to the very different levels of metal concentration. For example, Eibner et al.^11^ reported that high heavy metal loadings (2.5–7.1 wt %) in eucalyptus altered the pyrolysis decomposition pathway of cellulose, promoting the formation of 1-hydroxy-(1R)-3,6-dioxabicyclo[3.2.1]­octan-2-one and CO_2_. The pyrolysis of Pb naturally contaminated Avicennia marina^12^ resulted in a modest increase in the gas and char yields and a decrease in bio-oil yield, with all variations below 5 wt %. In contrast, the fast pyrolysis of water hyacinth biomass doped with lead nitrate^13^ resulted in a drastic decrease in the gas yield (15–30 wt % less than the control biomass, depending on the pyrolysis temperature) and a corresponding increase in the bio-oil yield (17–35 wt %); the char yield decreased slightly. However, the two studies differ in terms of both biomass contamination levels (40 ppm vs 2–10 wt %) and contamination nature (natural contamination or doping).
- Metal species-specific effects: the type of heavy metal and counterion strongly influence the liquid product composition. Pb and Cu were found to promote and inhibit, respectively, the formation of acetic acid.^13,14^ Moreover, Mayer et al.^15^ highlighted the influence of heavy metal (Zn and Pb) speciation as nitrates or sulfates on biomass decomposition: the addition of nitrates does not cause substantial variations in the decomposition rate of the doped biomass, while the sulfates greatly reduce the biomass reactivity.
- Influence of doping method: the choice of doping procedure should closely reproduce natural contamination conditions, as it can affect the reaction mechanisms during pyrolysis. Mayer et al.^15^ explored the effect of three different doping procedures (dry mixing, wet impregnation, and ion exchange), which resulted in different types of bindings between heavy metals and biomass tissues, and found milder effects than metal speciation. The three doping procedures resulted in a slight shift in the temperature of the maximum decomposition rate, ranging from 373 °C for dry mixing to 381 °C for ion exchange.

The aim of this work is to investigate the catalytic effect of Pb on poplar pyrolysis since Pb is one of the most widespread heavy metals in contaminated soils^16^ and poplar is one of the most abundant SRC.^17^ To our knowledge, this is the first systematic study assessing the combined effects of Pb speciation and contamination procedure on product yields (biochar, bio-oil, and gas) and characteristics at two pyrolysis temperatures relevant to bio-oil and biochar production (465 and 600 °C).^18^ To simulate different contamination scenarios, three doping procedures were applied to demineralized poplar biomass using lead acetate trihydrate (Pb­(CH_3_COO)_2_·3H_2_O, which will be referred to as PbAc) as the Pb source, namely, ion exchange, dry mixing, and wet impregnation. Dry mixing^15^ simulates the deposition of heavy metals on the outer layer of the biomass, without any chemical bond formation (detrital contamination^19^). Ion exchange^15^ simulates the chemical bonding between heavy metals and biomass components (authigenic contamination^19^). Wet impregnation^15^ simulates a mixture of authigenic and detrital contaminations, which is the usual occurrence in naturally contaminated biomass. Then, to evaluate the effect of Pb chemical speciation, plain poplar biomass was doped with PbAc and lead nitrate (Pb­(NO_3_)_2_, referred to as PbN) to simulate the presence of Pb in the biomass as an organic or inorganic salt.

## 2 Materials and Methods

### 2.1 Feedstock Preparation

Poplar biomass was treated to produce several pyrolysis feedstocks. Briefly, to investigate the effect of the contamination type, the biomass was demineralized and then doped with PbAc following different techniques.^20^ Demineralization was performed to isolate the effect of Pb bonding type, avoiding biases associated with the extensive washing required by the ion-exchange doping procedure. Without this step, differences among samples would partly arise from uncontrolled variations in native inorganic content. Predemineralization ensured comparable treatments across all samples, allowing the observed effects to be attributed specifically to Pb speciation and bonding. To investigate the effect of Pb chemical speciation, poplar biomass was doped with PbN and PbAc. In this case, untreated poplar was used as the parent biomass to better simulate real contaminated biomass. The complete procedure for feedstock preparation is described in detail in a previous work,^20^ and is reported in the Supporting Information. All of the feedstocks are listed in Table 1.

**Table 1:** List of Feedstock Names and Doping Procedures Adapted From ref^20,20^

parent biomass	Pb salt	doping method	feedstock name
demineralized poplar wood (**P_w**)	Pb(CH_3_COO)_2_	dry mixing	P_w+PbAc_dm
	Pb(CH_3_COO)_2_	ion exchange	P_w+PbAc_ie
	Pb(CH_3_COO)_2_	wet impregnation	P_w+PbAc_wi
untreated poplar wood (**P_p**)	Pb(NO_3_)_2_	wet impregnation	P_p+PbN_wi
	Pb(CH_3_COO)_2_	wet impregnation	P_p+PbAc_wi

The nominal Pb concentration of all doped feedstocks is 1000 ppm in the biomass. It should be noted that Pb concentrations on the order of 1000 ppm are generally reported for hyperaccumulator species^10^ and are not representative of Pb levels typically found in the woody tissues of nonhyperaccumulating plants, such as poplar. In this study, the selected Pb concentration was intentionally conservative and aimed at representing a worst-case scenario within the range of values reported for contaminated biomass across different species.

### 2.2 Feedstocks Characterization

The produced feedstocks are characterized as follows. The moisture, volatile, fixed carbon, and ash contents are obtained via proximate analysis, using a TGA 701 analyzer (LECO), following the standard ASTM D5142 method. The elemental compositions in terms of carbon (C), hydrogen (H), and nitrogen (N) are obtained using a CHN828 analyzer (LECO), and ethylenediaminetetraacetic acid (EDTA) as the calibration standard. Oxygen (O) is obtained by difference, considering the ash content obtained through proximate analysis.

To analyze the composition of the inorganic phase of the biomass, the organic phase of the sample is dissolved via microwave-assisted acid digestion according to the US-EPA 3051 method. The digested samples are then analyzed by using inductively coupled plasma mass spectrometry (ICP-MS) (7500CE, Agilent).

The thermal decomposition of the feedstocks is evaluated by using a Pyris STA 6000 thermogravimetric analyzer (PerkinElmer). The samples are heated in an inert atmosphere under a fixed N_2_ flux of 80 mL/min, reaching 800 °C at a heating rate of 10 °C/min. For each analyzed feedstock, the weight loss curve and differential thermogravimetry (DTG) curve, which represent the decomposition rate of the sample, are calculated.

### 2.3 Pyrolysis Experimental Setup

The pyrolysis system employed in the present work comprised two sections: the pyrolysis reactor and the exiting line, which can be arranged for either bio-oil collection or permanent gas analysis.

The reactor consists of a jacketed prismatic reaction chamber (*L* = 0.24 m, *W* = 0.04 m, *H* = 0.052 m), described in detail in previous works.^20,21^

The pyrolysis gas and condensable vapors exit the reactor, carried by the N_2_ flow, and are directed to the first cooling stage, which consists of a double-pipe heat exchanger that uses distilled water from a chiller as the cooling fluid. Then, the line changes according to the purpose of the pyrolysis test (Figure 1). Bio-oil collection and gas analysis are mutually exclusive: when collecting bio-oil, the low temperature induced by liquid nitrogen causes the solidification of CO_2_ in the trap, thus affecting the results of gas analysis.

**Figure 1:**
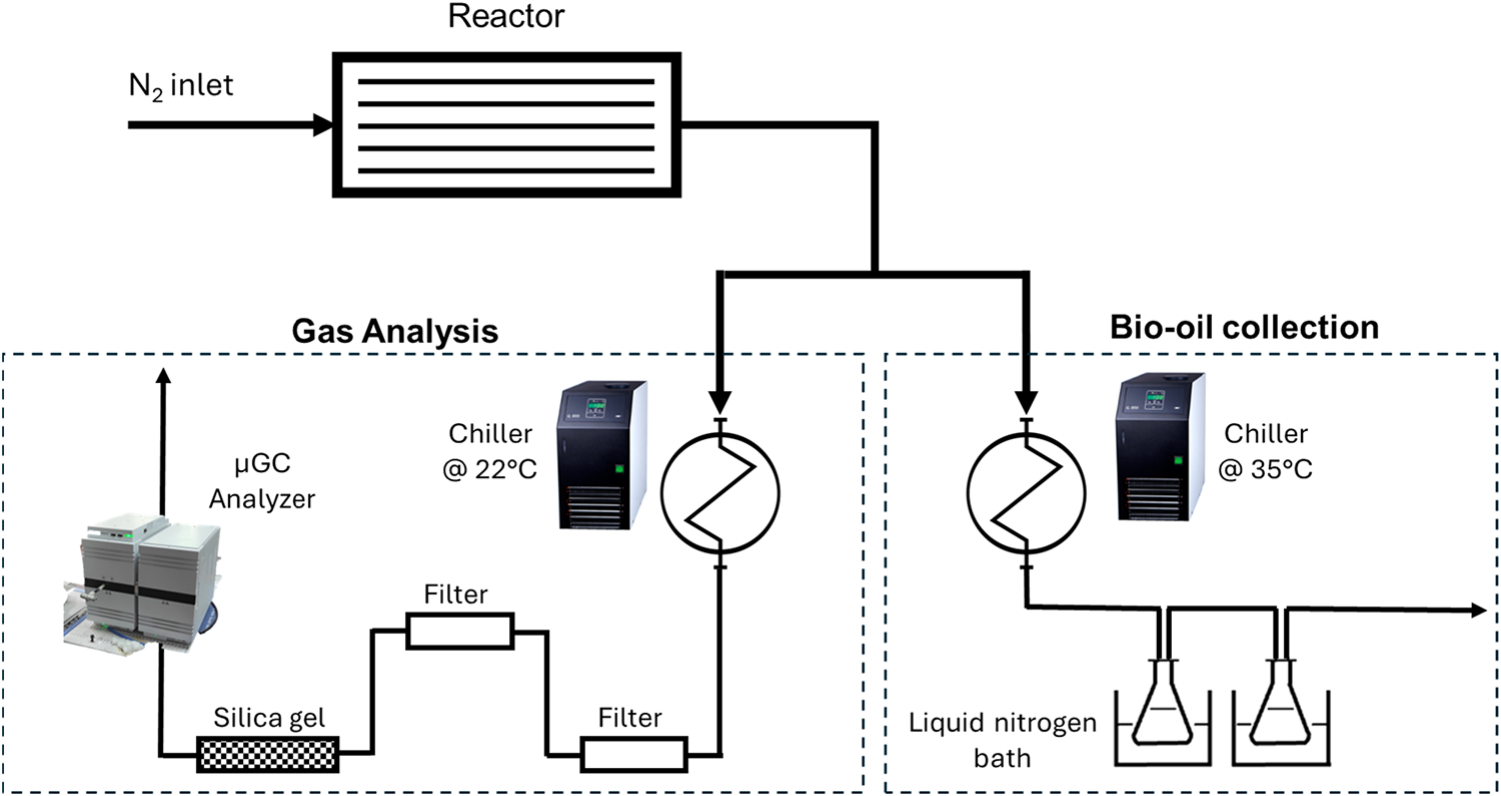
Schematic of the experimental apparatus scheme, representing the two exiting line configurations.

- Bio-oil collection: after the first heat exchanger, kept at 35 °C, the gas and vapors pass through a double condensation stage in liquid nitrogen, composed of two collection flasks immersed in a liquid nitrogen bath. After the double condensation stage, the gas flow is discharged directly.
- Pyrolysis gas analysis: after the first heat exchanger, the gas and vapors pass through a water-cooled glass condenser; both stages are maintained at 22 °C. After the condenser, the noncondensable gases are analyzed online using a μ-GC system after passing through two cotton filters and a silica gel trap for moisture removal and fine gas cleaning before the analysis.

All of the feedstocks were pyrolyzed at two different pyrolysis temperatures, 465 and 600 °C, which represent the limits of a typical temperature range of interest for bio-oil and biochar production. Moreover, 465 °C is reported to be the threshold temperature below which Pb is mostly retained in the char.^4,20,22^

The operational parameters other than pyrolysis temperature are kept constant throughout the experimental campaign and are as follows: mean heating rate = 5 °C/min; N_2_ flow rate = 3 NL/min; pressure = 1 atm. The pyrolysis tests and the derived pyrolysis products are indicated by the name of the feedstock, followed by the pyrolysis temperature.

### 2.4 Pyrolysis Product Characterization

#### 2.4.1 Biochar

The biochar yield is obtained by weighing the biochar and the biomass before and after the test, respectively; the reported yields are the average values of at least 2 tests. All of the produced biochar samples were characterized as follows:

- Elemental analysis was performed to determine the composition in terms of carbon (C), hydrogen (H), nitrogen (N), and oxygen (O) weight percentages, as described in Section 2.2.
- The ash content was determined by incinerating the samples in a muffle furnace at 700 °C for 2 h.
- Porosimetry was performed using a N_2_ adsorption porosimeter (Autosorb iQ, Quantachrome) after degassing the samples for 3 h at 200 °C. The surface area was determined according to the Brunauer–Emmett–Teller (BET) model.^23^

#### 2.4.2 Gas

Pyrolysis gas analysis was carried out online using a micro gas chromatography analyzer (Agilent 990 Micro GC) at a rate of one analysis every 125 s (corresponding to a temperature increase of about 10 °C). The quantified species corresponds to over 99% of the total produced gas.

The instrument is equipped with two chromatographic columns:

- A Molsieve 5A column, which is operated at 90 °C with argon as the gas carrier and separates the lighter gas species: H_2_, O_2_, N_2_, CH_4_, and CO.
- A PORAPLOT U column equipped with a PLOT Q precolumn is operated at 90 °C with helium as the gas carrier and separates the heavier gas species: CO_2_, C_2_H_4_, C_2_H_6_, C_2_H_2_, and C3.

Each column is followed by a TCD detector that measures the concentration of the species that exits the column. The gas analysis system was calibrated using specific gas mixtures containing known concentrations of H_2_, O_2_, N_2_, CO, CH_4_, CO_2_, C2, and C3.

The N_2_ flow was used to convert the volumetric composition of the gas phase into volumetric release rate curves of the gas species. By integrating the gas release rate, it was possible to calculate the produced gas volume (and mass) for each gas species. The data on C2 and C3 yields are omitted since the combined yield of these species was always below 0.15 wt % and therefore negligible compared to the total gas yield.

#### 2.4.3 Bio-Oil

The bio-oil yield is calculated as the difference. The bio-oil yield was estimated by weighing the collected bio-oil. However, not all of the bio-oil is successfully condensed in the cold traps; the heavier species can condense in a previous section of the outlet line or pyrolysis system, sticking to the tube walls, and the more volatile species can be entrained in the gas phase. The amount of bio-oil collected for the conducted pyrolysis tests was between 54% and 65% of the theoretically calculated bio-oil yield.

The bio-oil characterization was carried out as follows.

- Karl–Fischer Titration: the water content of the bio-oils was determined by using a Mettler Toledo V20 Karl Fischer volumetric titrator. The analysis was carried out according to the methodology reported in ASTM E203 using Aquastar Merck Chemicals; an amount of bio-oil between 8 and 20 mg was used for each analysis.
- The samples are analyzed using an Agilent 7890A gas chromatograph coupled with a mass spectrometer MS 5975C-VLMSD (GC-MS). The GC is equipped with a 624 capillary column, which operates with helium as the carrier gas. The bio-oil samples are highly diluted in acetone (10 wt %). 1 μL of each sample is injected for the analysis. The column is heated according to the following temperature program: 4 min at 45 °C, heating to 235 °C at a heating rate of 3 °C/min, and 50 min at 235 °C. The sample is injected in the “split mode” and carried by a flux of helium of 13.8 mL/min. The detector is set to scan a range of m/Z between 35 and 350; the lower limit was set to detect small species like acetic acid, while limiting the baseline noise. The bio-oil compounds are identified by matching the obtained spectra with the National Institute of Standards and Technology (NIST) library. The quantification of 24 representative compounds (2-Butanone, Glycolaldehyde, Acetic acid, 1-hydroxy-2-Propanone, Propanoic acid, Butanoic acid, Furfural, 2-Furanmethanol, 2-methyl-2-Cyclopenten-1-one, 1-(2-furanyl)-Ethanone, 2­(5H)-Furanone, 3-methyl-2-Cyclopenten-1-one, 3-methyl-1,2-Cyclopentanedione, Phenol, 2-methoxy-Phenol, p-Cresol, Creosol, 4-ethyl-2-methoxy-Phenol, 5-Hydroxymethylfurfural, 2-Methoxy-4-vinylphenol, 2,6-dimethoxy-Phenol, Hydroquinone, Vanillin, Levoglucosan) is carried out using the Agilent MassHunter Quantitative Analysis (Quant My-Way) software. The calibration curve for each compound is obtained with standard solutions in acetone at weight concentrations between 0.4% and 0.04% (except for acetic acid, whose calibration range is between 1.5% and 0.15%, glycolaldehyde, and 1-hydroxy-2-propanone, whose calibration range is between 1% and 0.1%); each calibration curve results in an *R* ^2^ index ≥ 0.98.

## 3 Results and Discussion

### 3.1 Feedstock Characterization

The results of the proximate and elemental analyses of the feedstocks are reported in Table 2 as weight percentages on a dry basis (db).

**Table 2:** Proximate and Elemental Analyses of the Feedstock Used in the Experimental Work Adapted from ref^20^

	volatile	ash	fixed carbon	C	H	N	O
	wt % d.b.						
P_w	82.9 (±0.2)	0.8 (±0.1)	16.4 (±0.1)	48.0 (±0.4)	6.1 (±0.1)	0.3 (±0.0)	44.8 (±0.7)
P_w+PbAc_dm	84.5 (±0.3)	0.4 (±0.3)	15.1 (±0.1)	48.0 (±0.4)	6.1 (±0.1)	0.3 (±0.0)	45.2 (±0.9)
P_w+PbAc_ie	85.3 (±0.3)	0.2 (±0.1)	14.4 (±0.3)	49.1 (±0.3)	6.2 (±0.1)	0.3 (±0.1)	44.2 (±0.6)
P_w+PbAc_wi	83.7 (±1.1)	0.6 (±0.1)	15.6 (±1.0)	48.0 (±0.7)	6.1 (±0.1)	0.5 (±0.5)	44.7 (±1.3)
P_p	81.8 (±0.9)	1.5 (±0.2)	16.7 (±0.6)	46.2 (±1.4)	6.1 (±0.1)	0.3 (±0.2)	46.0 (±1.9)
P_p+PbN_wi	81.3 (±0.2)	1.5 (±0.1)	17.1 (±0.1)	47.2 (±0.4)	6.1 (±0.3)	0.2 (±0.2)	44.9 (±0.9)
P_p+PbAc_wi	80.9 (±0.7)	1.5 (±0.4)	17.6 (±0.7)	47.9 (±0.2)	6.1 (±0.0)	0.5 (±0.1)	43.9 (±0.6)

Volatiles account for more than 80% of the total weight on a dry basis for all the feedstocks, while the ash content is quite low, even for a woody biomass:^24^ P_p, and the derived doped feedstocks have an ash content of 1.5%. The demineralization procedure was effective with P_w and the derived feedstocks retaining about half the amount of ash originally contained in P_p. There is no other appreciable effect on the biomass composition, either for the different doping procedures or for Pb speciation.

The analysis of the inorganic fraction of the feedstocks, expressed in terms of weight concentration, is reported in Table 3.

**Table 3:** Concentration of the Main Inorganics Species of the Feedstock used in the Experimental Work Adapted from ref^20^

mg/kg	P_w	P_w+PbAc_dm	P_w+PbAc_ie	P_w+PbAc_wi	P_p	P_p+PbN_wi	P_p+PbAc_wi
Mg	216 (±15)	273 (±9)	315 (±40)	292 (±58)	408 (±30)	506 (±28)	510 (±116)
P	78 (±21)	55 (±9)	43 (±9)	63 (±13)	512 (±11)	512 (±124)	556 (±40)
K	240 (±117)	330 (±35)	78 (±10)	288 (±58)	1649 (±98)	1734 (±356)	2155 (±420)
Ca	1991 (±392)	1991 (±122)	2523 (±269)	2023 (±405)	1738 (±276)	2742 (±285)	4488 (±1506)
Pb	9 (±2)	878 (±21)	1147 (±117)	787 (±157)	9 (±1)	890 (±105)	767 (±11)

As for the Pb content, it must be noted that the doping procedure is affected by the doping efficiency. Therefore, the actual concentration of Pb in the biomass is not easily predictable and can differ from the target concentration (1000 ppm). The actual Pb concentration of each sample was measured after the doping procedure, resulting in a slight variability among the doped samples, but in all cases, 2 orders of magnitude higher than that of the control samples. The efficiency of the demineralization procedure is confirmed: in fact, the content of Mg is halved in P_w with respect to P_p, while the P and K contents are reduced to 15% of the initial content. On the other hand, Ca was not affected by demineralization; this behavior has already been observed for the water demineralization of poplar wood chips.^25^ The Pb content naturally occurring in the biomass was also unaffected by the demineralization procedure, as confirmed by the values obtained for the P_p and P_w feedstocks. The P_w+PbAc_ie feedstock has a considerably lower content of K than the other demineralized feedstocks, indicating that the ion-exchange procedure also acts as a further demineralization step.

Ultimately, from the feedstock characterization, it was found that there were no relevant differences induced by doping the biomass, except for the Pb content and speciation. Thus, the modification in the pyrolysis product yields and properties can be attributed to the presence of Pb in various forms.

Figure 2 shows the weight loss and differential thermogravimetric (DTG) curves of the doped feedstocks and reference untreated biomasses. The only feedstock that was not analyzed by TG analysis is P_w+PbAc_dm, as it was not possible to obtain homogeneous samples through dry mixing at such a low weight scale.

**Figure 2:**
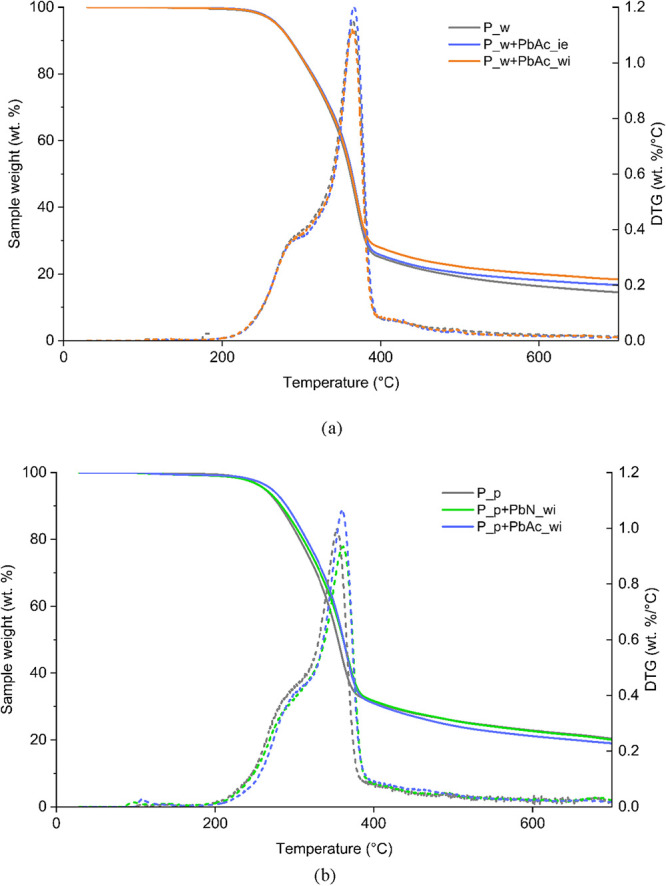
Weight loss and differential thermogravimetric (DTG) curves of (a) P_w and derived doped feedstocks and (b) P_p and derived doped feedstocks.

The different doping methods (Figure 2a) led to slight variations in the solid residue after the thermal treatment. More specifically, the variations in both the maximum devolatilization rate and the solid residue fall within the experimental uncertainty, which can be mainly attributed to the heterogeneity of the biomass when working with such small sample quantities. The effect of alkali and alkali-earth metals (AAEMs), which is well documented in the literature,^8^ is also clearly visible by comparing P_w (Figure 2a) and P_p (Figure 2b): the presence of AAEMs causes an increase in the solid residue of the decomposition, a decrease in the maximum decomposition rate, and a shift of this maximum toward lower temperatures.

Regarding the effect of Pb chemical speciation, neither P_p+PbN_wi nor P_p+PbAc_wi exhibited remarkably different weight loss curves from P_p (Figure 2b). However, there are some mild effects on the maximum decomposition temperatures. In both cases, the presence of Pb salts results in a shift in the maximum decomposition rate to higher temperatures, about 10 °C higher than that of P_p. The use of different salts in wet impregnation induces the presence of both cations and anions in the aqueous medium, and the acid–base properties of the anions must be considered due to their possible effect on the biomass structure and thus on the pyrolytic biomass behavior. These effects have already been observed for the inherent AAEMs present in lignocellulosic biomasses.^26^ For example, NO_3_^–^ exhibited higher catalytic activity than HPO_3_^2–^,^27^ while CH_3_CO_2_^–^ was found to have a lower activity than the corresponding CO_3_^2–^. However, these results can be affected by the lower concentration of acetate on a molar basis.^28^

The interpretation of the TGA results requires some caution for two main reasons. First, this technique provides information only on the solid yield (i.e., the residual biochar) without quantifying the relative proportions of gaseous and condensable products. Second, because of the very small sample size typically used in thermogravimetric analysis, the intrinsic heterogeneity of the feedstock may not be fully captured, and bulk phenomena that occur when larger biomass quantities are processed during pyrolysis may be overlooked. Therefore, the product yields obtained from the experimental plant, shown in Figure 3, should also be considered.

**Figure 3:**
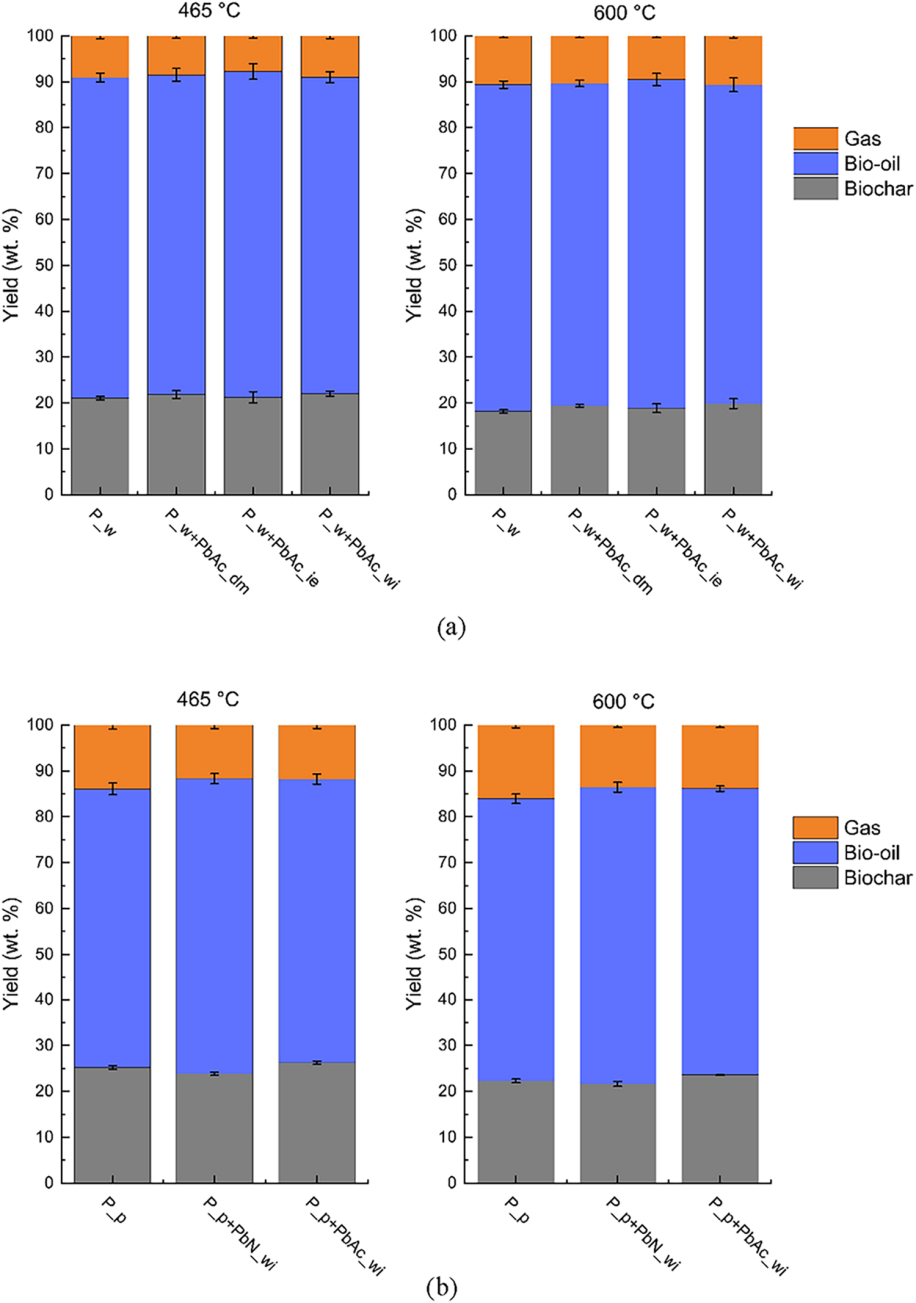
Pyrolysis product yield at different temperatures of (a) P_w and derived doped feedstocks and (b) P_p and derived doped feedstocks.

### 3.2 Product Yield

Figure 3 shows the mean values of the pyrolysis product yields, namely, biochar, bio-oil, and permanent gas yields. Each pyrolysis test was repeated at least twice.

For each feedstock, the biochar yield decreases as the pyrolysis temperature increases, ranging from 21–26 wt % at 465 °C to 18–23.5 wt % at 600 °C. Bio-oil yields are almost independent of pyrolysis temperature, while gas yields increase from 465 to 600 °C, as expected. There is a strong effect of the inherent inorganics removed via demineralization: for both of the considered temperatures, P_p (and derived doped biochars) have higher biochar and gas yields and lower bio-oil yield compared to P_w (and derived doped biochars). Considering the effect of the doping procedure, at 465 °C, the biochar yield of all of the doped samples is comparable to that of P_w. As the pyrolysis temperature increased to 600 °C, all of the demineralized doped samples resulted in a slightly higher biochar yield than P_w. By comparing P_w+PbAc_wi and P_w+PbAc_ie, the results of the thermogravimetric analysis are partially confirmed: the sample doped via wet impregnation has a biochar yield slightly higher than that doped via ion exchange. However, the results of the pyrolysis tests are affected by higher variability than those of the thermogravimetric analysis. Different Pb speciation also causes slight differences in the biochar yield at 465 °C, with P_p+PbN_wi resulting in a lower yield and P_p+PbAc_wi resulting in a higher yield than P_p. At 600 °C, P_p and P_p+PbN_wi have comparable yields, while P_p+PbAc_wi still has a slightly higher biochar yield. The variation in the bio-oil yield is more relevant: P_p+PbN_wi results in a higher bio-oil yield than P_p (65% wt. against 60% wt.), while P_p+PbAc_wi and P_p have comparable bio-oil yields. Both of the Pb salts have comparable effects on gas production, causing a decrease in the gas yield with respect to P_p. All the observed yield variations are more pronounced for the nondemineralized feedstocks, indicating possible interactions between Pb and inherent inorganics that are removed during demineralization.

The variation in the pyrolysis product yields has also been reported in the literature: Pb affects product yields, but the extent of yield variation is related to the Pb concentration, contamination specifics, and biomass composition. For instance, the impregnation of water hyacinth with a high quantity of PbN (Pb content 2–10 wt %)^13^ resulted in only a slight decrease of the biochar yield when compared with the untreated biomass, while bio-oil and gas yields showed higher variations. The PbN addition causes a decrease in the gas yield and an almost corresponding increase in the bio-oil yield over the whole considered temperature range (275–550 °C), as also observed in the present study. Similar results were obtained for Pb nitrate impregnated *Arundo donax* ^29^ pyrolyzed at 450 °C. The introduction of PbN caused an increase in the bio-oil yield at the expense of gas and biochar yields for *Arundo donax*. However, the same authors observed an increase in both the bio-oil and gas yields for Broussonetia papyrifera compared to untreated biomass.

### 3.3 Biochar Properties

The biochar composition is reported in terms of carbon (C), hydrogen (H), nitrogen (N), and oxygen (O) weight percentages in Table 4. The ash content is also reported in the same table.

**Table 4:** Elemental Analysis of the Biochars Produced at Different Temperatures Adapted from ref^20^

	C	H	N	O (a)	Ash
sample	wt % d.b.				
*T* = 465 °C					
P_w	76.4 (±0.3)	3.4 (±0.1)	0.5 (±0.0)	16.1 (±0.4)	3.6
P_w+PbAc_dm	77.4 (±0.4)	3.3 (±0.0)	0.3 (±0.1)	17.2 (±0.5)	1.8
P_w+PbAc_ie	76.6 (±0.3)	3.4 (±0.0)	0.1 (±0.0)	19.0 (±0.3)	1.0
P_w+PbAc_wi	77.2 (±0.2)	3.4 (±0.0)	0.2 (±0.1)	16.5 (±0.3)	2.8
P_p	77.2 (±0.7)	3.1 (±0.0)	0.7 (±0.2)	13.4 (±1.0)	5.7
P_p+PbN_wi	74.6 (±0.6)	3.3 (±0.0)	0.8 (±0.1)	15.0 (±0.7)	6.4
P_p+PbAc_wi	75.0 (±0.7)	3.2 (±0.0)	0.5 (±0.1)	15.4 (±0.8)	5.8
*T* = 600 °C					
P_w	83.2 (±1.0)	2.6 (±0.0)	0.4 (±0.1)	9.7 (±1.2)	4.2
P_w+PbAc_dm	84.8 (±0.5)	2.5 (±0.0)	0.4 (±0.0)	10.3 (±0.5)	2.1
P_w+PbAc_ie	85.3 (±0.6)	2.6 (±0.0)	0.1 (±0.1)	10.8 (±0.7)	1.2
P_w+PbAc_wi	84.0 (±0.5)	2.5 (±0.1)	0.3 (±0.1)	10.2 (±0.6)	3.1
P_p	83.8 (±1.4)	2.3 (±0.0)	0.9 (±0.1)	6.6 (±1.4)	6.4
P_p+PbN_wi	80.1 (±1.7)	2.2 (±0.1)	1.0 (±0.2)	9.7 (±2.0)	7.0
P_p+PbAc_wi	82.5 (±0.5)	2.4 (±0.1)	0.4 (±0.1)	8.2 (±0.7)	6.4

A common trend is observed for all feedstocks: as the pyrolysis temperature increases, the C content of the biochar increases from an average of 76% at 465 °C to 83.5% at 600 °C, while the H and O contents decrease from 3.3% and 16.1% to 2.4% and 9.3%, respectively. The observed variations in the N content are within the experimental error. A direct comparison with literature data for the nitrogen content is difficult, as the reported results are often contrasting due to both the variability in the original biomass and the chemical nature of the nitrogen species involved. This implies that there is no clear trend with temperature for lignocellulosic biomass, whereas a decreasing trend was observed for N-rich feedstocks, such as sewage sludge.^30^ Samples derived from demineralized biomass have a generally higher O content that is related to their lower ash content.^8^ To investigate the effect of Pb speciation and bonding with biomass, the C and O contents of chars in response to temperature change and the presence of Pb were represented as the O/C and H/C molar ratios on a dry ash-free basis in the Van Krevelen diagram shown in Figure 4.

**Figure 4:**
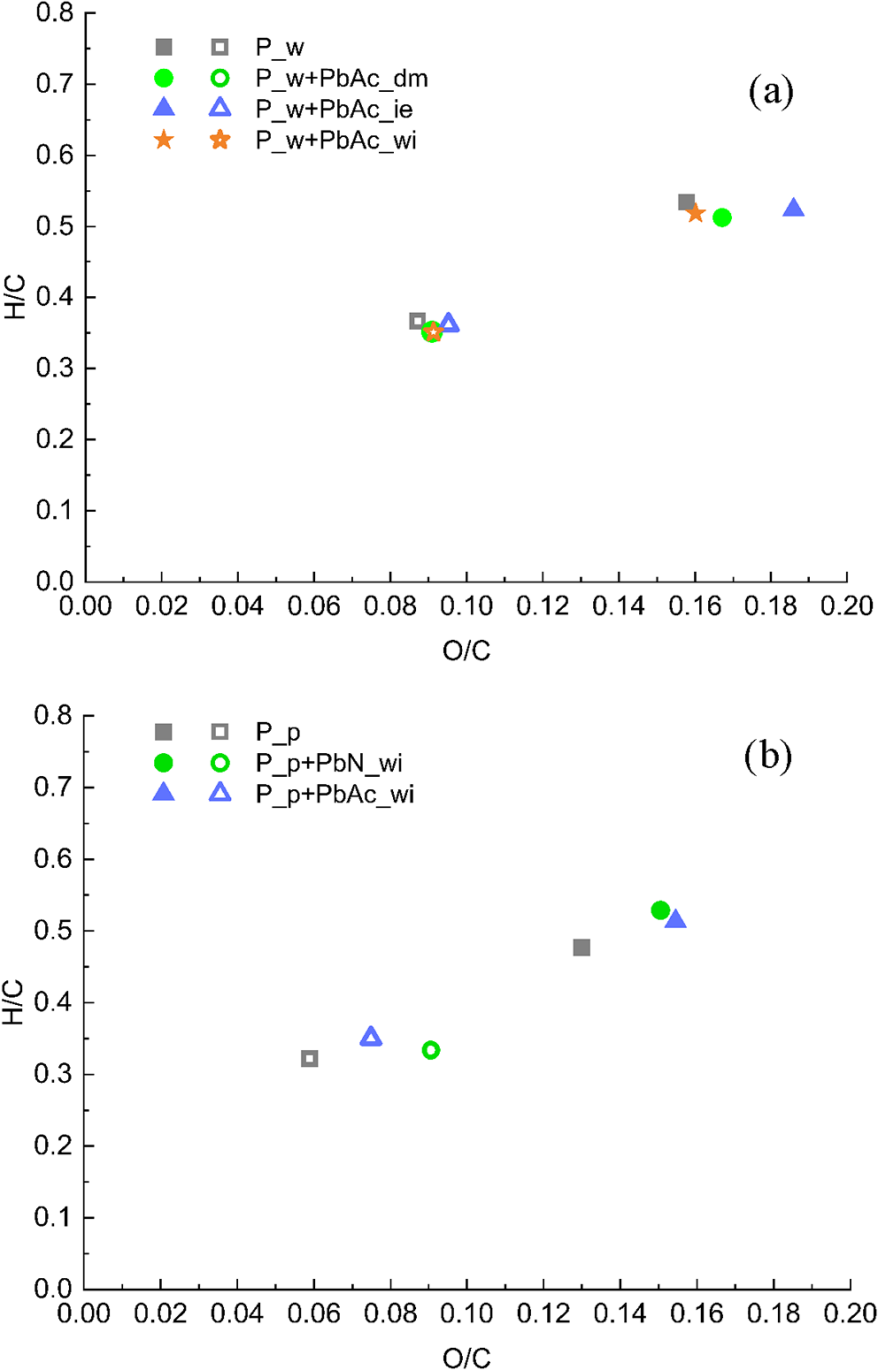
H/C and O/C ratios of biochars produced from (a) P_w and derived doped feedstocks and (b) P_p and derived doped feedstocks. Full symbols refer to 465 °C and open symbols refer to 600 °C.

The chars produced at 465 °C are the result of the cleavage of the glycosidic linkages between the sugars of cellulose and of dehydration and depolymerization reactions (removal of carbonyl, carboxyl, and hydroxyl groups) and their C, O and H contents meet the typical values observed for other biomasses, resulting in a decrease of both O/C and H/C ratios with respect to the initial feedstocks (ranging between 0.68–0.75 and 1.50–1.58, respectively). As the temperature increases, the carboxyl, carbonyl, and methoxyl groups are released, and they completely fall off at temperatures in the range of 600–700 °C. As expected from the elemental analysis, the H/C and O/C ratios decrease with temperature for all the considered biochars.

Considering the effect of Pb bonding with biomass tissues, no relevant differences can be observed between P_w and the derived doped samples. The slightly higher O content of P_w + PbAc_ie at 465 °C is attributed to its lower ash content due to the further inorganic removal in the final washing step of the ion-exchange doping procedure.

The introduction of different Pb salts exerts a pronounced influence on the compositional evolution of the solid residue. While both doped samples display the same monotonic decrease in the H/C and O/C ratios with increasing temperature, their O/C values consistently exceed those of the undoped biochar (P_p). This systematic divergence indicates that the presence of Pb hampers the devolatilization of O-bearing functional groups, thereby limiting the O release of the oxo into the vapor phase across the entire investigated temperature domain. The attenuated reduction in the O/C ratio is consistent with the lower overall gas yield shown in Figure 3 and, more specifically, with the reduced CO_2_ formation observed in Table 5. Since CO_2_ exhibits an intrinsically high O/C ratio compared with most other condensable species, its diminished evolution preferentially retains oxygen in the solid phase, producing biochar with a relatively higher oxygen content and lower carbon depletion with respect to P_p. This behavior is most pronounced for P_p+PbN_wi, which attains higher O/C ratios at 600 °C than its acetate-doped counterpart. This result is in agreement with the literature findings obtained with other metal (Ni, Zn, Cu, and Co) acetates.^31^ To assess whether this effect persists at higher temperatures, the O/C ratios of the biochars produced from both doped and undoped P_p at 800 °C were evaluated, and the results confirmed that the phenomenon remained evident. Indeed, from 600 to 800 °C, the O/C ratio of doped biochar does not decrease further and is considerably higher than that of the undoped biochar P_p (0.09, 0.08, and 0.04 for P_p+PbN_wi, P_p+PbAc_wi, and P_p, respectively).

**Table 5:** Effect of the Doping Procedure and Pb Salt on Gaseous Species Yields (wt %) at Different Temperatures

	H_2_	CH_4_	CO	CO_2_
	wt.%			
465 °C				
P_w	0.01 (±0.00)	0.36 (±0.00)	2.48 (±0.04)	6.26 (±0.41)
P_w+PbAc_dm	0.01 (±0.00)	0.33 (±0.00)	2.39 (±0.04)	5.77 (±0.38)
P_w+PbAc_ie	0.01 (±0.00)	0.33 (±0.00)	2.16 (±0.04)	5.26 (±0.34)
P_w+PbAc_wi	0.01 (±0.00)	0.34 (±0.00)	2.59 (±0.05)	6.06 (±0.40)
P_p	0.02 (±0.00)	0.35 (±0.00)	3.32 (±0.06)	10.29 (±0.67)
P_p+PbN_wi	0.02 (±0.00)	0.36 (±0.00)	3.19 (±0.06)	8.11 (±0.53)
P_p+PbAc_wi	0.01 (±0.00)	0.35 (±0.00)	3.35 (±0.06)	8.31 (±0.54)
600 °C				
P_w	0.07 (±0.00)	0.69 (±0.03)	3.24 (±0.02)	6.83 (±0.42)
P_w+PbAc_dm	0.05 (±0.00)	0.66 (±0.02)	3.30 (±0.02)	6.34 (±0.39)
P_w+PbAc_ie	0.07 (±0.00)	0.62 (±0.02)	3.04 (±0.02)	5.85 (±0.36)
P_w+PbAc_wi	0.08 (±0.00)	0.64 (±0.02)	3.48 (±0.03)	6.59 (±0.41)
P_p	0.11 (±0.00)	0.75 (±0.03)	4.13 (±0.03)	11.22 (±0.69)
P_p+PbN_wi	0.12 (±0.00)	0.71 (±0.03)	4.02 (±0.03)	8.76 (±0.54)
P_p+PbAc_wi	0.09 (±0.00)	0.68 (±0.03)	4.08 (±0.03)	8.98 (±0.55)

The divergent behavior exhibited by P_p+PbAc_wi and P_w+PbAc_wi with respect to their undoped counterparts (P_p and P_w) indicates that the inhibitory role of Pb on O devolatilization during pyrolysis becomes pronounced only in the presence of a higher concentration of co-occurring inorganics. For instance, phosphorus is known to promote oxygen retention in the solid phase by forming thermally stable phosphate and polyphosphate species and stabilizing oxygen-containing functional groups through P–O–C linkages, thereby reducing their conversion into gaseous products. The presence of Pb in a P-rich environment can further enhance this effect through the formation of highly refractory Pb–O–P structures, which strengthen the immobilization of oxygen within the carbon matrix as hydroxyl and carboxylic groups.^32^ This evidence points to a cooperative immobilization mechanism, in which Pb acts as a catalytic/structural cofactor within a phosphorus-dominated inorganic framework.^33,34^ Also, other inherent inorganics, such as K and Mg, have similar effects on the biochar O content when coupled with Pb. Indeed, these inorganics contribute to increasing Pb stability^20,35^ through a mechanism likely analogous to that reported above for P,^36^ causing an increase of the O content in the biochar. This aspect warrants further investigation to elucidate the specific coordination environments involved and to quantify the relative contributions of each inorganic component.

The effects of both the doping modality and the nature of the Pb salt used during pyrolysis propagate into the physicochemical evolution of poplar, ultimately translating into distinct modifications of the porous structure of the resulting char. The development of the porosity of the biochars is shown as a BET specific surface area in Figure 5. As expected, increasing the pyrolysis temperature has a beneficial effect on the BET surface area. At low temperatures, all biochars exhibit very limited surface development, and no appreciable differences are observed among the samples, as the emergence of porosity is primarily associated with the release of volatiles and the subsequent reorganization of the carbon matrix, processes that become significant only at higher temperatures.

**Figure 5:**
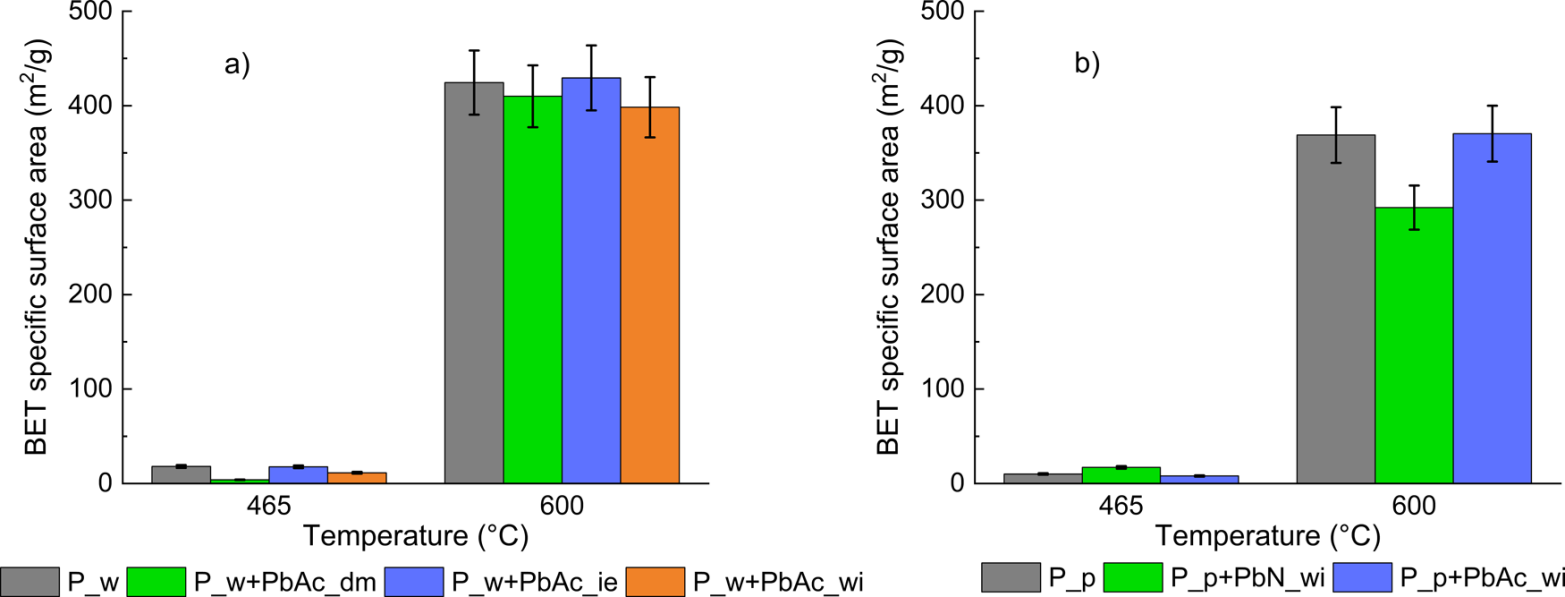
BET specific surface area of biochars produced from (a) P_w and derived doped feedstocks and (b) P_p and derived doped feedstocks.

The BET surface area is higher at 600 °C, and since the removal of AAEMs results in an increase of the volatile fraction, the demineralized samples, either doped or untreated, have a higher BET surface area than P_p.^37^ Consistent with that observed for the bulk chemistry, the doping procedure itself does not influence the BET surface area of the resulting biochars. However, the nature of the Pb salt plays a decisive role: PbN exerts a detrimental effect on pore development, whereas PbAc does not induce any appreciable variation compared to the undoped P_p sample. To the best of our knowledge, no studies have reported BET surface area measurements for wood-derived biochars doped with Pb (either nitrate or acetate). Han et al.^29^ did not observe any strong interaction of Pb with the biomass components, resulting in a qualitatively similar porosity development of the doped and untreated biomasses based on scanning electron microscopy (SEM) observations. Heavy metals were found to be uniformly distributed in the pore structure of the chars. However, SEM analysis cannot detect differences at the nanoscale, which is the dimension scale of the micro- and mesopores that mostly contribute to the BET surface.

Pb speciation affects porosity development, as the addition of PbN to P_p has a detrimental effect on the BET, while the addition of PbAc does not have a relevant effect. The negative effect of Pb nitrate cannot be ascribed to the occurrence of melting and sintering phenomena of the salts because at this temperature, both PbN and PbAc are totally decomposed,^38,39^ leaving a solid residue of PbO that has a melting temperature higher than 600 °C. Nevertheless, the literature on inorganic doping and secondary reactions of pyrolysis vapors provides consistent mechanistic evidence that explains the observed decrease in porosity through pore blocking^40^ and secondary char (coke) formation and deposition phenomena.^41^ The effect of the counterion on pore development must be invoked to explain the difference observed between P_p+PbN_wi and P_p+PbAc_wi. For instance, the nitrate (NO_3_^–^) anion has been found to generate a biochar characterized by a lower BET surface area with respect to other counterions (OH^–^ and CO_3_^2–^) when associated with K.^42^ However, to the authors’ knowledge, these kinds of studies are not available for heavy metals and are scarcely available even for inherent inorganics.

### 3.4 Gas Composition

Table 5 shows the yields of the single gaseous species. The main released permanent gases are CO_2_ and CO, with yields in the ranges of 6–11 wt % and 2–4 wt %, respectively, depending on the feedstock and pyrolysis temperature. The CH_4_ and H_2_ yields are lower, especially for H_2_, whose yield reaches a maximum of 0.1 wt % due to the low highest temperature investigated in the present study.^43,44^ To provide a more complete description of gas production during pyrolysis, the normalized gas release curves are reported in the Supporting Information, Figure S1.

The use of different doping procedures does not cause great variations in the gas species’ yields. The most pronounced effect is the decrease of both the CO and CO_2_ yields for P_w+PbAc_ie, at both 465 and 600 °C, when compared to the reference biomass P_w. However, this effect can be mostly attributed to the further decrease in the AAEM content of the sample doped via ion exchange. In fact, the presence of AAEMs favors the production of CO and CO_2_, a phenomenon that is well documented in the scientific literature and is also confirmed by comparing the results of P_p and P_w in the present work. The different bonding of Pb with the biomass tissues, however, does not affect the shape of the release curves of CO and CO_2_ (Figure S1), with the highest gas production between 250 and 370 °C. On the other hand, the presence of inherent inorganics causes a shift in the maximum release rate of both CO and CO_2_ toward lower temperatures, which increases from 330–335 °C for P_p (and derived doped samples) to 350 °C for P_w (and derived doped samples).

The effect is further corroborated by the differences observed in samples doped with different Pb salts, with both P_p+PbN_wi and P_p+PbAc_wi, resulting in a considerable reduction in the CO_2_ yield when compared to P_p. The CO_2_ yield reduction is more abundant for nondemineralized doped feedstocks than for P_w-derived doped feedstocks, suggesting that Pb has a greater effect in the presence of inherent AAEMs. As reported in the literature, Pb inhibits the production of CO_2_ released from the decomposition of cellulose by stabilizing the carbonyl and carboxyl groups,^13^ whose cracking is the main source of CO_2_ production. This phenomenon appears more relevant for P_p+PbN_wi, which, despite having roughly the same CO_2_ yield as P_p+PbAc_wi, has a peculiar CO_2_ release curve; this is the only sample that does not show any shoulder of the main release peak between 250 and 300 °C (Figure S1).

The presence of Pb does not result in other modifications of the shape of the release curves of the permanent gases or in shifts of the characteristic temperatures, except for that described above.

### 3.5 Bio-Oil Composition

Figure 6 shows the water yields of the produced bio-oils. Water yield is primarily influenced by the type of contamination, whereas temperature and specific contaminant species play comparatively minor roles. More specifically, at low temperatures, PbAc acts as a catalyst for the dehydration reactions of the holocellulosic fraction^45^ when added as a molecular species through dry mixing. Conversely, when Pb is introduced into biomass via ion exchange, it exerts a detrimental effect on water production. These two opposing effects tend to compensate for each other when Pb is added through the wet impregnation procedure, as this method results in the simultaneous presence of Pb both as PbAc molecules deposited on the external surface and as ion-exchanged species.

**Figure 6:**
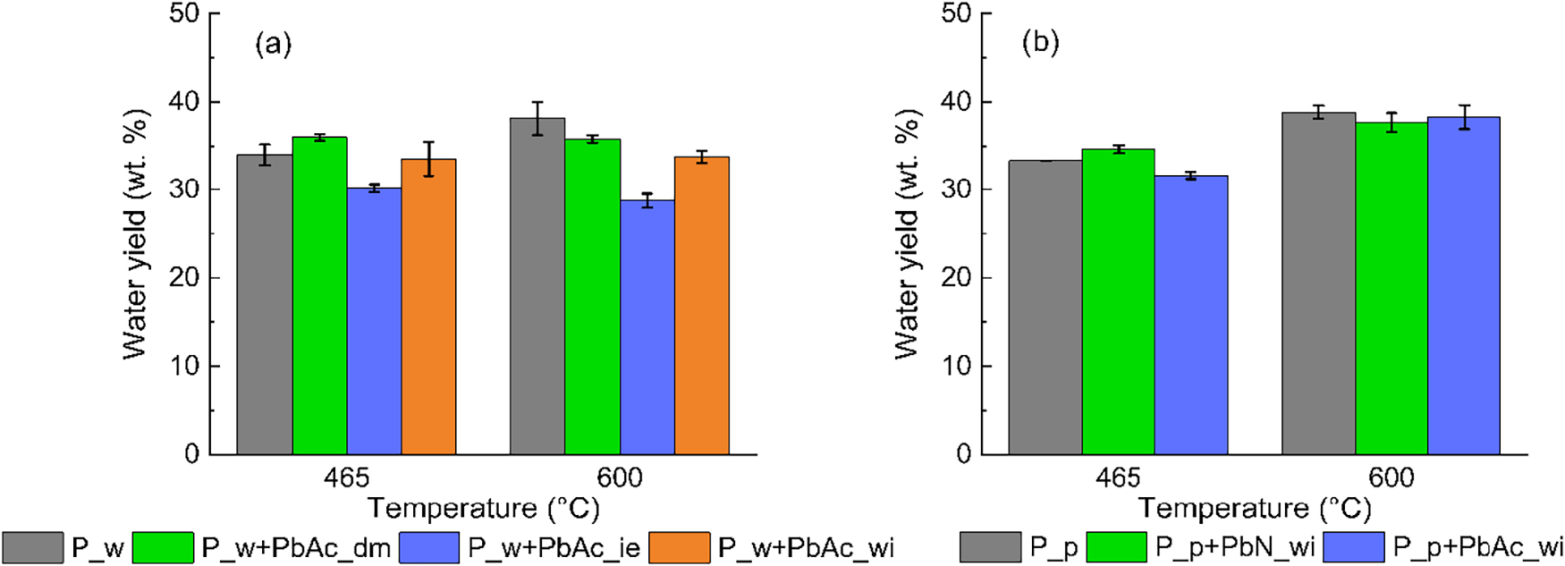
Water yields for (a) P_w and derived doped feedstocks and (b) P_p and derived doped feedstocks.

At higher temperatures, water is mainly generated through the dehydration reactions of the lignin fraction.^46^ At 600 °C, the presence of Pb appears to inhibit lignin dehydration reactions, which instead proceed in the case of the Pw sample, leading to an increase in the water yield from 34 to 38.1 wt %.

Different Pb salts do not cause significant changes at high temperatures. Interestingly, in these cases, the presence of Pb does not suppress lignin dehydration, neither in the form of PbAc nor PbN. It can be inferred that the presence of alkali and alkaline earth metals (AAEMs) can hinder the Pb effects, as discussed in Section 3.6 On the other hand, at 465 °C, the sample doped with PbAc results in a slightly lower water yield, confirming the difference observed in the case of the demineralized samples. The sample doped with PbN exhibits a different behavior, resulting in a slight increase in the water yield at low temperatures, thus confirming that different Pb salts interact differently with the biomass during the initial stages of the pyrolysis process.

Figure 7 shows the yields of the bio-oil compound classes resulting from the GC-MS analyses. Given the complexity of the bio-oil composition, the 24 quantified compounds are grouped into seven classes: carboxylic acids, furans, sugars, ketones, aldehydes, phenols, guaiacols, and syringols. The full characterization of the bio-oils is reported in Tables S2 and S3 of the Supporting Information, together with the composition of the selected compound classes. In general, the pyrolysis temperature has little influence on the yields of the bio-oil classes, in agreement with the constant total bio-oil yield in this temperature range (Figure 3). The only exception is carboxylic acids (mainly acetic acid, accounting for 95–98% of the total carboxylic acids), whose yield shows an increase of about 2% from 465 to 600 °C in the case of the P_p sample doped with PbAc through wet impregnation. It is likely that this effect is due to the simultaneous presence of inherent AAEMs and Pb, as the same effect is not observed in the P_w plain and doped samples.

**Figure 7:**
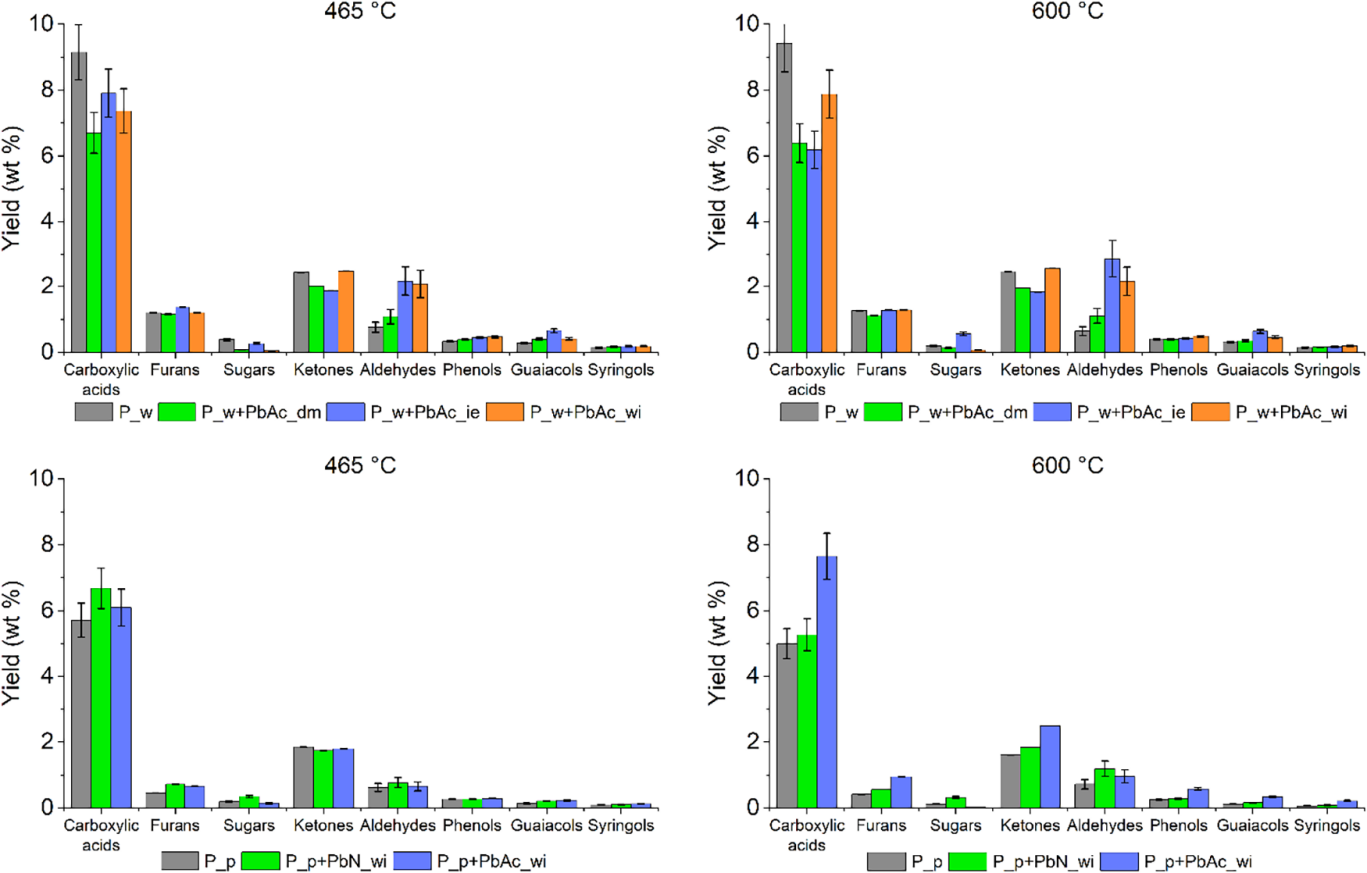
Yields of bio-oil compound classes.

The presence of Pb influences the formation of carboxylic acids in different ways depending on the inorganic content of the starting feedstock. Specifically, the yield of carboxylic acids decreases in the Pb-doped P_w samples, regardless of the doping procedure employed. In contrast, for P_p-doped samples, Pb does not exert any significant effect at low temperatures; however, at high temperatures, the presence of PbAc leads to an increased yield of carboxylic acids, in opposition to the trend observed for the corresponding Pb-doped P_w samples under the same conditions. This behavior suggests the combined effect of the inherent inorganics and Pb should be considered to explain the observed results, as will be further discussed in Section 3.6.

In the case of aldehydes (glycolaldehyde) and phenols (phenols, guaiacols, and syringols), P_w+PbAc_ie and P_w+PbAc_wi show a greater increase in the yield than P_w+PbAc_dm with respect to P_w at both pyrolysis temperatures. This indicates that while the presence of Pb favors the production of these compounds during biomass pyrolysis, the Pb salt deposited on the outer layer of the biomass has a lower effect than Pb ions bonded to biomass tissues. The positive effect of PbAc on the yield of aldehydes and phenols is confirmed by comparing P_p and the derived doped feedstocks: P_p+PbAc_wi has a higher yield of these compounds at both 465 and 600 °C. However, the yield increase for aldehydes is lower in this case than for the demineralized feedstocks, probably due to the presence of a higher concentration of AAEMs. Although AAEMs also have a positive effect on aldehyde production,^47^ their high concentration could overshadow the Pb effects, resulting in an overall lower yield increase. Aldehyde production is also influenced by Pb speciation: in fact, P_p+PbN_wi causes a higher increase in the glycolaldehyde yield than P_p+PbAc_wi (0.8–1.2% and 0.6–1%, with respect to 0.6–0.7% for P_p). A similar behavior is observed for sugars, namely levoglucosan. This observation suggests that PbN is more prone to interaction with the cellulose and hemicellulose decomposition pathways than PbAc, causing less severe cracking reactions and favoring the formation of such species at the expense of gas species such as CO and CO_2_.

### 3.6 Inherent Inorganics Role

To clarify the possible interactions and synergistic effects between Pb and inherent AAEMs, a detailed comparison is carried out among samples characterized by different AAEM contents but subjected to the same doping procedure (wet impregnation with PbAc). This approach allows for the isolation of the combined role of Pb and inherent inorganics, highlighting effects that are not observable when Pb or AAEMs are present individually at low concentrations.

Overall, the results (summarized in the Supporting Information, Figures S2–S5) indicate that the impact of Pb strongly depends on the inherent AAEM content, with different behaviors emerging for different product properties.

#### 3.6.1 Effect on Biochar O/C Ratio and CO_2_ Yield

For samples with low AAEM content (P_w and P_w+PbAc_wi), the presence of Pb does not induce appreciable modifications. As shown in Figure S2, both doped and undoped samples follow the same temperature-dependent trend of decreasing O/C ratio from about 0.16 at 465 °C to approximately 0.09 at 600 °C, whereas CO_2_ yield (Figure S3) remains almost unaffected by Pb addition at both 465 and 600 °C. In contrast, when considering the samples richer in AAEMs (P_p and P_p+PbAc_wi), the presence of Pb had noticeable effects. The O/C ratio, while following the same decreasing trend with temperature as observed for the demineralized samples, its absolute value increases in the presence of Pb, increasing from 0.13 to 0.155 at 465 °C and from 0.06 to 0.075 at 600 °C (Figure S2). Conversely, the CO_2_ yield decreases in the presence of Pb, from approximately 10–11 wt.% for P_p in the range of 465 °C–600 °C to 8–9 wt.% for P_p+PbAc_wi in the same temperature range (Figure S3). The observed variations could be ascribed to the inhibitory effect of Pb on the decarboxylation and decarbonylation reactions of the holocellulose fraction, catalyzed by AAEMs.

#### 3.6.2 Effect on the Water Yield

The influence of Pb on water formation also depends on the AAEM content. For the AAEM-rich sample (P_p), the addition of Pb has a negligible effect on the water yield (Figure S4). Conversely, for samples with a low AAEM content, a significant reduction in water yield from 38.1 to 33.8 wt % can be observed at 600 °C. At this temperature, it can be postulated that Pb has an inhibitory effect on lignin dehydration reactions. Such an effect is not noticeable if more AAEMs are present in the biomass because of the well-documented catalytic role of inherent inorganics in promoting lignin dehydration, which prevails over the inhibitory effect of Pb.

#### 3.6.3 Effect on the Carboxylic Acid Yield

The presence of Pb causes opposite effects on the yields of carboxylic acids (Figure S5) depending on the AAEM content. For the samples with low AAEM content, Pb induces a decrease of the carboxylic acid yield from 7.4–7.9 wt.% to 7.9 wt % to 9.1 to 9.4 wt % in the considered pyrolysis temperature range. This indicates that the presence of Pb could hinder the formation of carboxylic acids. Conversely, for AAEM-rich samples (P_p and P_p+PbAc_wi), Pb promotes carboxylic acid production, particularly at high temperatures: at 600 °C, P_p+PbAc_wi reaches about 7.6 wt % compared to roughly 5 wt % for P_p.

Overall, it can be observed that while Pb alone can affect the properties of the pyrolysis product, the presence of AAEMs and other inherent inorganics can result in significant synergistic effects with Pb, altering both the magnitude and direction of Pb-induced changes.

## 4 Conclusions

This work investigates the effects of Pb contamination on the slow pyrolysis of lignocellulosic biomass, focusing on the effects of Pb bonding type, Pb chemical speciation, and inherent inorganic content (AAEMs). Different Pb–biomass interactions were simulated through controlled doping procedures, and Pb speciation effects were assessed by using lead acetate and lead nitrate. Overall, the results show that Pb alters the pyrolysis product distribution and properties, with effects that are strongly dependent on the inorganic matrix of the biomass. In particular:

- The properties of biochar are affected mainly by the chemical speciation of Pb, with PbN leading to a higher O/C ratio and lower BET surface area compared to PbAc. Different Pb bonding has a negligible impact when the AAEM content is low, but it is amplified in AAEM-rich biomasses, indicating synergistic interactions.
- The effect of Pb on the formation of permanent gases is strongly controlled by the presence of AAEMs in the feedstock. CO_2_ inhibition is weakly affected by Pb speciation or bonding, but is strongly enhanced by the presence of AAEMs.
- The yields of carboxylic acids, aldehydes, and sugars are affected by Pb, with trends strongly modulated by AAEM content. In demineralized biomasses, Pb generally suppresses carboxylic acid formation, whereas in AAEM-rich biomasses, the simultaneous presence of Pb and AAEMs can reverse this trend, enhancing carboxylic acid yields at higher temperatures.

Overall, these results clearly demonstrate that the effects of Pb cannot be interpreted independently of the inorganic biomass matrix, highlighting the need for systematic studies to elucidate contaminant–AAEM interactions during the pyrolysis of contaminated biomass.

## Supplementary Material



## Notes

Feedstocks preparation procedure; release rates of the measured gas species; O/C ratio, CO_2_ yield, water yield, and carboxylic acid yield of the samples doped with PbAc through wet impregnation and of the reference samples; and detailed GC-MS analysis results (PDF)
