# Supplementary material for: Influence of Pb Bonding and Speciation on the Pyrolysis Products of Contaminated Biomass
Source: Energy Fuels. 2026 Feb 14;40(8):4229–41. doi: 10.1021/acs.energyfuels.5c05968 (PMC12951667; doi:10.1021/acs.energyfuels.5c05968)
Supplement: Supplementary file 1 [file ef5c05968_si_001.pdf]

Supporting Information

Influence of Pb bonding and speciation on pyrolysis products of contaminated biomass

Davide Amato[a], *Paola Giudicianni*[a*], *Corinna Maria Grottola*[a], *Raffaele Ragucci*[a].

[a]Istituto di Scienze e Tecnologie per l'Energia e la Mobilità Sostenibili (STEMS-CNR), Via Marconi 4, 80125, Naples, Italy

Feedstocks preparation

To test the influence of Pb contamination type, poplar biomass (P_p) was demineralized to avoid bias due to the removal of inherent inorganics in the further step of the ion exchange procedure. Demineralization was obtained by washing P_p with distilled water: 100 g of biomass were soaked in 2 L of distilled water and continuously stirred for roughly 70 hours at room temperature. The biomass was then filtered, rinsed with 2 L of distilled water and kept in oven at 80 ˚C until completely dry. The obtained demineralized biomass, that will be referred to as P_w, was doped following the three procedures described below using lead acetate tri-hydrate as doping salt (salt purity ≥ 99.8%). Lead acetate was employed as doping salt to simulate heavy metals bound to organic acids of the biomass; moreover, among the organic acids, acetates have a higher solubility in water[1].

- Dry mixing[2]: 0.025 g of salt were mechanically mixed for at least 15 minutes with 16 g of demineralized biomass. The produced feedstock is referred to as P_w+PbAc_dm, and it simulates detrital contamination.
- Ion exchange[2]: 0.24 g of salt were dissolved in 2L of distilled water; 100 g of demineralized biomass were then added to the solution and stirred continuously for 100 hours at room temperature. After 100 hours the biomass was filtered, rinsed with 2 L of distilled water and immersed in 2 L of distilled water; after 72 hours of continuous stirring at room temperature the biomass was filtered again and dried in oven at 80 ˚C. The produced feedstock is referred to as P_w+PbAc_ie, and it simulates authigenic contamination.
- Wet impregnation[2]: 0.16 g of salt were dissolved in 2L of distilled water; 100 g of demineralized biomass were then added to the solution, stirred for 1 hour at room temperature and kept in oven at 80 ˚C until the complete evaporation of the water. The

produced feedstock is referred to as P_w+PbAc_wi, and it simulates a mixture of authigenic and detrital contamination.

A sample of P_w+PbAc_wi was treated with the same washing procedure described for ion exchange and was then analyzed to determine the resulting Pb concentration. 75 % of the original Pb content was still present in the biomass due to ion exchange mechanism (authigenic contamination). The remaining 25 % of Pb was removed by washing (detrital contamination).

To elucidate the influence of Pb chemical speciation on the pyrolysis process, P_p was doped via wet impregnation[2] using lead acetate tri-hydrate and lead nitrate (salt purity ≥99.9%) through the same procedure described before. Lead nitrate was considered as inorganic Pb salt because of its high solubility and the scarce influence of the nitrate counter-ion on the pyrolysis process.[3,4]

Two solutions were prepared by dissolving Pb salts in distilled water; 100 g of plain biomass was then added to the solution, stirred for 1 hour and kept in oven at 80 °C until the complete evaporation of the water. In the following, the lead acetate doped biomass and the lead nitrate doped biomass are identified as P_p+PbAc_wi and P_p+PbN_wi, respectively.

References

(1) Koppolu, L.; Clements, L. D. Pyrolysis as a Technique for Separating Heavy Metals from Hyperaccumulators. Part I: Preparation of Synthetic Hyperaccumulator Biomass. *Biomass Bioenergy* 2003, *24* (1), 69–79. https://doi.org/10.1016/S0961-9534(02)00074-0.

(2) Mayer, Z. A.; Apfelbacher, A.; Hornung, A. Effect of Sample Preparation on the Thermal Degradation of Metal-Added Biomass. *J Anal Appl Pyrolysis* 2012, *94*, 170–176. https://doi.org/10.1016/j.jaap.2011.12.008.

(3) Collard, F. X.; Blin, J.; Bensakhria, A.; Valette, J. Influence of Impregnated Metal on the Pyrolysis Conversion of Biomass Constituents. *J Anal Appl Pyrolysis* 2012, *95*, 213–226. https://doi.org/10.1016/J.JAAP.2012.02.009.

(4) Trubetskaya, A.; Lange, H.; Wittgens, B.; Brunsvik, A.; Crestini, C.; Rova, U.; Christakopoulos, P.; Leahy, J. J.; Matsakas, L. Structural and Thermal Characterization of Novel Organosolv Lignins from Wood and Herbaceous Sources. *Processes 2020, Vol. 8, Page 860* 2020, *8* (7), 860. https://doi.org/10.3390/PR8070860.

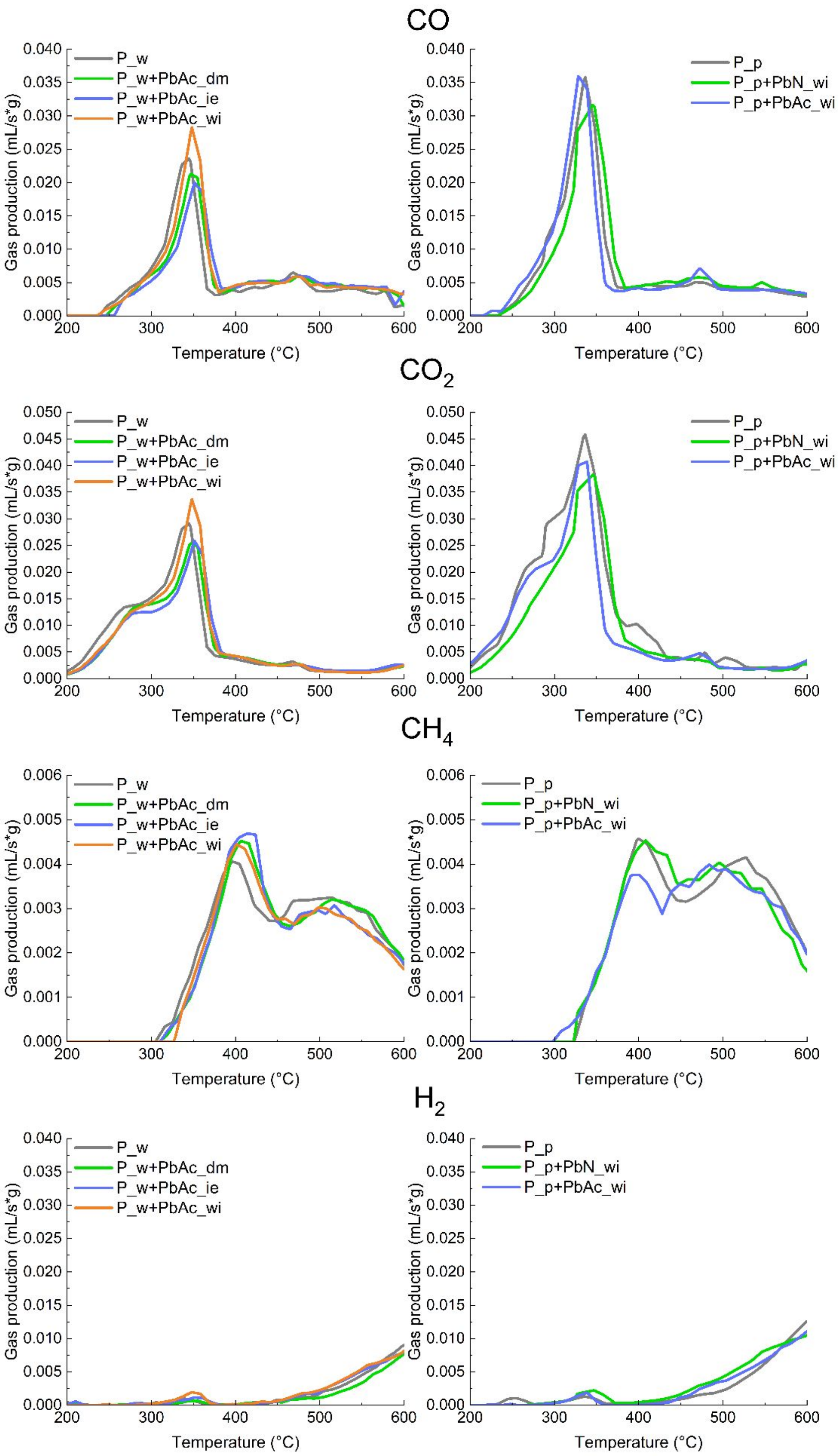

Figure S1 – Normalized production rates of the permanent gas species for all the doped and untreated feedstocks

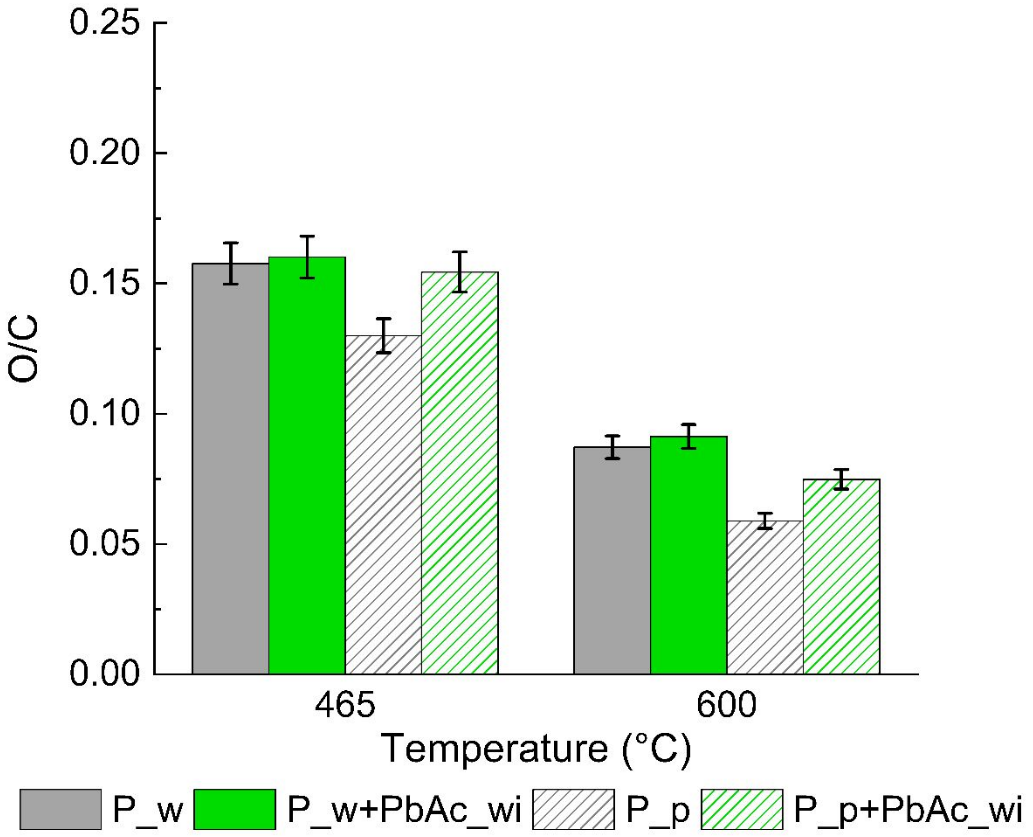

Figure S2 – O/C ratio of the samples doped with PbAc through wet impregnation and of the reference samples

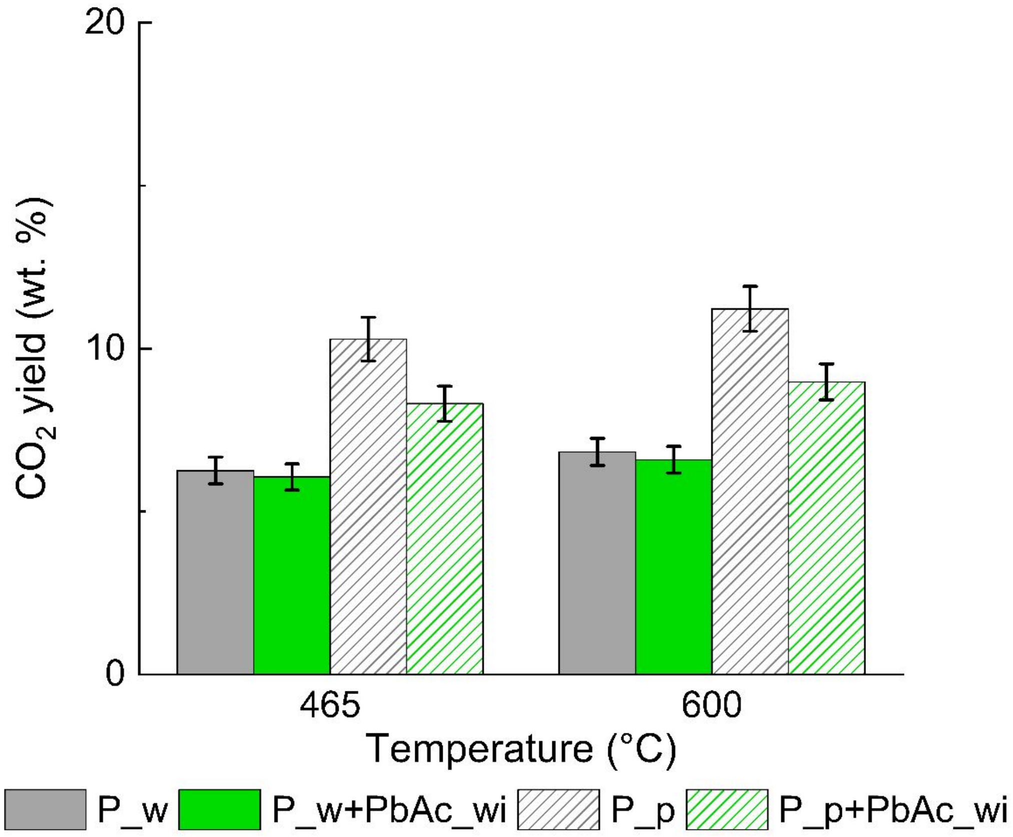

Figure S3 – CO_2 yield of the samples doped with PbAc through wet impregnation and of the samples feedstocks

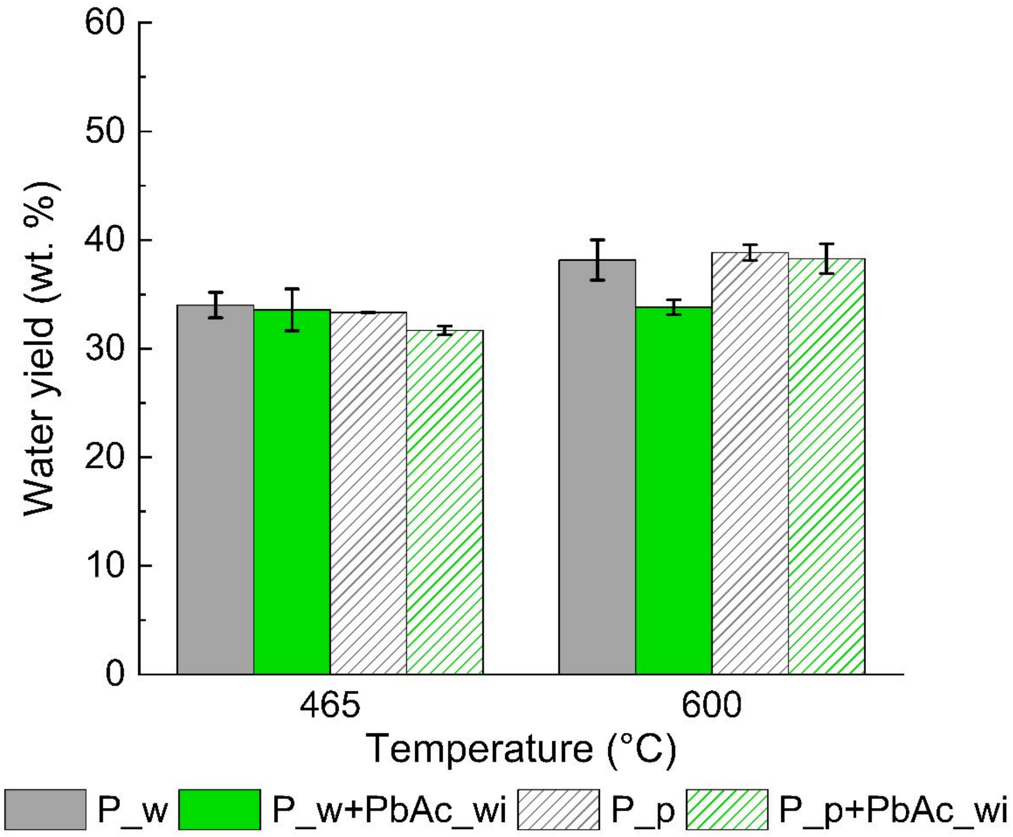

Figure S4 – Water yield of the samples doped with PbAc through wet impregnation and of the reference samples

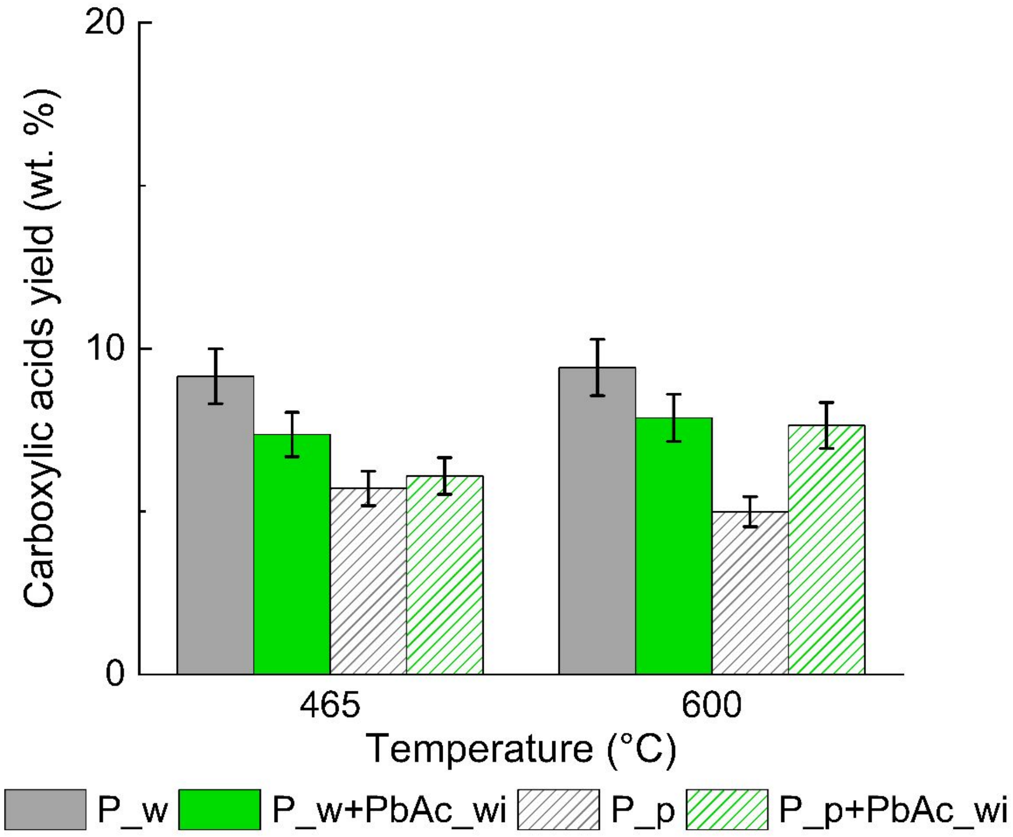

Figure S5 – Carboxylic acids yield of the samples doped with PbAc through wet impregnation and of the reference samples

Table S1 - Pyrolysis products yield at different temperatures.

	Biochar yield, wt. %	Bio-oil yield, wt. %	Gas yield, wt. %
**T=465 ˚C**			
P_w	21.1 (±0.4)	69.8 (±1.0)	9.1 (±0.6)
P_w+PbAc_dm	21.8 (±0.9)	69.7 (±1.4)	8.5 (±0.5)
P_w+PbAc_ie	21.3 (±1.2)	71.0 (±1.7)	7.8 (±0.5)
P_w+PbAc_wi	22.0 (±0.6)	69.0 (±1.2)	9.0 (±0.6)
P_p	25.2 (±0.4)	60.9 (±1.3)	13.90 (±0.9)
P_p+PbN_wi	23.8 (±0.3)	64.5 (±1.1)	11.68 (±0.7)
P_p+PbAc_wi	26.3 (±0.4)	61.9 (±1.1)	11.80 (±0.8)
**T=600 ˚C**			
P_w	18.2 (±0.4)	71.2 (±0.8)	10.6 (±0.4)
P_w+PbAc_dm	19.4 (±0.3)	70.3 (±0.7)	10.3 (±0.4)
P_w+PbAc_ie	18.9 (±0.9)	71.6 (±1.3)	9.5 (±0.4)
P_w+PbAc_wi	19.9 (±1.1)	69.5 (±1.5)	10.7 (±0.4)
P_p	22.3 (±0.4)	61.7 (±1.0)	16.04 (±0.6)
P_p+PbN_wi	21.6 (±0.6)	64.8 (±1.1)	13.55 (±0.5)
P_p+PbAc_wi	23.6 (±0.1)	62.6 (±0.6)	13.84 (±0.5)

Table S2 – Yield of single compounds in the bio-oils produced at 450 ˚C from all the doped and untreated feedstocks

465 ˚C							
	P_w	P_w+PbAc_dm	P_w+PbAc_ie	P_w+PbAc_wi	P_p	P_p+PbN_wi	P_p+PbAc_wi
Carboxylic acids							
Acetic acid	8.994	6.560	7.783	7.225	5.560	6.564	5.928
Propanoic acid	0.139	0.122	0.106	0.121	0.135	0.097	0.131
Butanoic acid	0.016	0.015	0.014	0.016	0.014	0.012	0.033
Furans							
Furfural	0.507	0.389	0.494	0.395	0.194	0.282	0.200
2-Furanmethanol	0.110	0.182	0.171	0.202	0.008	0.149	0.179
1-(2-furanyl)-Ethanone	0.016	0.016	0.017	0.018	0.015	0.014	0.019
2(5H)-Furanone	0.315	0.320	0.289	0.368	0.203	0.219	0.217
5-Hydroxymethylfurfural	0.259	0.253	0.404	0.222	0.035	0.058	0.039
Sugars							
Levoglucosan	0.389	0.082	0.269	0.052	0.194	0.347	0.147
Ketones							
2-Butanone	0.008	0.024	0.034	0.027	0.025	0.034	0.034
1-hydroxy- 2-Propanone	2.306	1.853	1.675	2.287	1.737	1.636	1.670

2-methyl-2-Cyclopenten-1-one	0.021	0.019	0.022	0.019	0.017	0.017	0.020
3-methyl-2-Cyclopenten-1-one	0.016	0.016	0.016	0.016	0.014	0.012	0.015
3-methyl-1,2-Cyclopentanedione	0.077	0.103	0.123	0.117	0.058	0.047	0.066
Aldheydes							
Glycolaldehyde	0.762	1.084	2.166	2.078	0.621	0.770	0.655
Phenols							
Phenol	0.296	0.351	0.416	0.431	0.235	0.240	0.245
p-Cresol	0.021	0.021	0.019	0.018	0.013	0.014	0.017
Hydroquinone	0.018	0.013	0.011	0.017	0.022	0.017	0.030
Guaiacols							
2-methoxy-Phenol	0.103	0.112	0.115	0.117	0.077	0.076	0.094
Creosol	0.081	0.097	0.119	0.095	0.029	0.040	0.038
4-ethyl-2-methoxy-Phenol	0.013	0.020	0.022	0.020	0.007	0.008	0.011
2-Methoxy-4-vinylphenol	0.061	0.146	0.370	0.148	0.010	0.076	0.080
Vanillin	0.024	0.024	0.034	0.028	0.011	0.010	0.009
Syringols							

2,6-dimethoxy-Phenol	0.148	0.161	0.182	0.186	0.094	0.105	0.129

Table S3 – Yield of single compounds in the bio-oils produced at 600 ˚C from all the doped and untreated feedstocks

600 ˚C							
	P_w	P_w+PbAc_dm	P_w+PbAc_ie	P_w+PbAc_wi	P_p	P_p+PbN_wi	P_p+PbAc_wi
Carboxylic acids							
Acetic acid	9.262	6.262	6.074	7.718	4.876	5.152	7.321
Propanoic acid	0.141	0.110	0.093	0.139	0.107	0.100	0.270
Butanoic acid	0.017	0.014	0.014	0.018	0.012	0.013	0.052
Furans							
Furfural	0.520	0.367	0.402	0.410	0.158	0.201	0.237
2-Furanmethanol	0.132	0.166	0.178	0.219	0.004	0.007	0.292
1-(2-furanyl)-Ethanone	0.017	0.014	0.017	0.020	0.014	0.014	0.028
2(5H)-Furanone	0.354	0.312	0.297	0.410	0.206	0.273	0.339
5-Hydroxymethylfurfural	0.247	0.261	0.388	0.227	0.029	0.070	0.051
Sugars							
Levoglucosan	0.206	0.133	0.566	0.065	0.118	0.324	0.018
Ketones							

2-Butanone	0.029	0.023	0.031	0.034	0.014	0.009	0.031
1-hydroxy- 2-Propanone	2.296	1.790	1.631	2.344	1.523	1.737	2.272
2-methyl-2-Cyclopenten-1-one	0.022	0.018	0.022	0.022	0.014	0.015	0.027
3-methyl-2-Cyclopenten-1-one	0.017	0.016	0.019	0.020	0.013	0.015	0.024
3-methyl-1,2-Cyclopentanedione	0.087	0.101	0.129	0.134	0.050	0.068	0.136
Aldheydes							
Glycolaldehyde	0.645	1.108	2.861	2.157	0.722	1.193	0.963
Phenols							
Phenol	0.359	0.348	0.372	0.440	0.221	0.262	0.510
p-Cresol	0.032	0.027	0.031	0.027	0.016	0.010	0.031
Hydroquinone	0.014	0.016	0.012	0.018	0.015	0.016	0.040
Guaiacols							
2-methoxy-Phenol	0.114	0.105	0.123	0.133	0.071	0.084	0.151
Creosol	0.090	0.090	0.125	0.109	0.025	0.036	0.057
4-ethyl-2-methoxy-Phenol	0.014	0.018	0.022	0.024	0.005	0.007	0.019
2-Methoxy-4-vinylphenol	0.059	0.117	0.336	0.166	0.009	0.014	0.093

Vanillin	0.022	0.025	0.029	0.026	0.010	0.013	0.016
Syringols							
2,6-dimethoxy-Phenol	0.147	0.157	0.172	0.196	0.067	0.084	0.213
